# Next-Generation Sequencing of Aquatic Oligochaetes: Comparison of Experimental Communities

**DOI:** 10.1371/journal.pone.0148644

**Published:** 2016-02-11

**Authors:** Régis Vivien, Franck Lejzerowicz, Jan Pawlowski

**Affiliations:** 1 Swiss Centre for Applied Ecotoxicology (Ecotox Centre), Eawag/EPFL, 1015, Lausanne, Switzerland; 2 Department of Genetics and Evolution, University of Geneva, Geneva, Switzerland; Queensland University of Technology, AUSTRALIA

## Abstract

Aquatic oligochaetes are a common group of freshwater benthic invertebrates known to be very sensitive to environmental changes and currently used as bioindicators in some countries. However, more extensive application of oligochaetes for assessing the ecological quality of sediments in watercourses and lakes would require overcoming the difficulties related to morphology-based identification of oligochaetes species. This study tested the Next-Generation Sequencing (NGS) of a standard cytochrome c oxydase I (COI) barcode as a tool for the rapid assessment of oligochaete diversity in environmental samples, based on mixed specimen samples. To know the composition of each sample we Sanger sequenced every specimen present in these samples. Our study showed that a large majority of OTUs (Operational Taxonomic Unit) could be detected by NGS analyses. We also observed congruence between the NGS and specimen abundance data for several but not all OTUs. Because the differences in sequence abundance data were consistent across samples, we exploited these variations to empirically design correction factors. We showed that such factors increased the congruence between the values of oligochaetes-based indices inferred from the NGS and the Sanger-sequenced specimen data. The validation of these correction factors by further experimental studies will be needed for the adaptation and use of NGS technology in biomonitoring studies based on oligochaete communities.

## Introduction

Aquatic oligochaetes are abundant in fine, sandy and coarse sediments of watercourses and lakes, as well as in the hyporheic zones and groundwaters and include a large number of species characterized by a wide range of pollution sensitivity [1–3]. Since 1960, oligochaetes have been used in many countries for assessing the ecological quality of watercourses and lakes [4–7].

Several biotic indices based on the analysis of oligochaete assemblages have been proposed to assess the quality of sediments of watercourses and lakes [3]. The Oligochaete Index of Sediment Bioindication (IOBS), applied in the present study, allows the assessment of the quality of fine/sandy sediments of watercourses [8, 9]. For some years, this index has been applied in the canton of Geneva (Switzerland) as part of the program of quality monitoring of watercourses of the Water Ecology Service [10].

The difficult taxonomic identification of aquatic oligochaetes based on morphological features has compromised the more common use of this taxocenosis for eco-diagnostic analyses. Most species of the sub-family Tubificinae and the families Lumbriculidae and Enchytraeidae cannot be identified in an immature state, while they may represent over 80% of the specimens in a sample. In addition, species identification of the vast majority of Lumbriculidae and Enchytraeidae requires practicing dissection, which is unrealizable in routine analyses. Furthermore, various molecular studies have revealed the existence of cryptic species within aquatic oligochaetes [11–15], undetectable with the morphological approach.

DNA barcoding allows the rapid identification of a species by matching the sequence of a selected gene to a reference library and thus can overcome the issues associated with morphological identification [16]. The application of DNA barcoding to identify oligochaete species would facilitate their use in biomonitoring studies and allow the improvement of ecological diagnostics. Many studies show that the mitochondrial COI gene is a very effective barcode for aquatic and terrestrial oligochaetes [3, 17–20]. Since 2012, a database from COI sequences of aquatic oligochaetes collected in Switzerland has been initiated [20]. A 10% threshold in COI divergence was suggested for segregating between congeneric species in this group [20–22].

Recently, NGS-based metabarcoding has been proposed as a cost effective way to overcome the limitations of morphological identification in routine biomonitoring, allowing more efficient and reliable surveys [23–27]. Main efforts have been directed towards (i) checking the efficiency of DNA barcodes to identify important bioindicator taxa [16, 28–30], (ii) evaluating methods of sample preservation and DNA isolation [25, 31, 32], and (iii) testing the recovery of the structure and composition of artificial samples made with mixed specimens [28, 33–36]. The taxonomic groups that have been examined in most detail are the arthropods, particularly the chironomids and other aquatic insects [16, 31, 33], and the diatoms [29, 30].

Our study is the first attempt to apply NGS-based COI metabarcoding to analyse a community of aquatic oligochaetes. We tested the accuracy of the Illumina/Miseq technology in recovering the composition and abundance of controlled oligochaete communities by comparing the NGS data obtained from mixed specimen samples with the reference data obtained after Sanger sequencing of single specimens composing these samples. We also calculated the IOBS index both with the data obtained with Sanger sequenced specimens and NGS in order to determine whether the variations in composition and abundance of OTUs obtained with the NGS approach can have an influence on IOBS index value.

## Material and Methods

### Sampling of oligochaete specimens and mixed sample preparation

Freshwater sediments were sampled in two watercourses of the canton of Geneva (Switzerland), Seymaz and Avril. The sediments of the Seymaz river were sampled at Claparède in October 2013 (Site 1: 46.18849°N 6.18508°E) and at De Haller in March 2014 (Site 3: 46.20112°N 6.19612°E). The sediments of the Avril river were sampled at Bourdigny in January 2014 (Site 2: 46.21660°N 6.04665°E). The Water Ecology Service of the State of Geneva issued the permission to conduct this study on these sites.

At the laboratory, the sediment samples were fixed in ethanol (final volume of 95–100%) and then sieved through a column of 5 mm and 0.5 mm mesh size sieves. A total of 113, 110 and 107 oligochaetes specimens were sorted from sites 1, 2 and 3, respectively. The specimens sorted from site 1 were split into three sub-samples: 43 specimens (sample 1), 42 specimens (sample 2) and 28 specimens (sample 3). The specimens from site 2 were split into two sub-samples: 78 specimens (sample 4) and 32 specimens (sample 5). The 107 specimens sorted from site 3 were not split (sample 6). We split the samples of sites 1 and 2 in order to compare NGS and Sanger approaches on samples composed of different numbers of specimens.

The posterior region of each specimen (330 specimens in total) was cut transversally and divided into two parts. One part was used for Sanger sequencing in order to obtain the COI sequence of each specimen. The other part was used for NGS analysis and the parts of all specimens of a given sample were pooled (six mixed samples). In each mixed sample, special care was taken to keep more or less the same quantity of tissue between parts in order to limit the impact that different biomasses could have on the number of NGS sequences. The anterior parts of specimens were preserved in ethanol.

### DNA extraction, PCR and Sanger sequencing of oligochaete specimens

The total genomic DNA of the 330 oligochaete specimens were extracted using the guanidine thiocyanate method described by Tkach & Pawlowski [37]. A fragment of 658 base pairs of the COI gene was amplified using LCO 1490 and HCO 2198 primers [38]. Each PCR was performed in a total volume of 20 μl containing 0.6 Unit of Taq polymerase (Roche), 2 μl of the 10X buffer (Roche) containing 20 mM of MgCl_2_, 0.5 μl of each primer (10 mM each), 0.4 μl of a mix containing 10 mM of each dNTP (Roche) and 0.8 μl of template DNA of undetermined concentration. The PCR process was comprised of an initial denaturation step at 95°C for 5 min, followed by 35 cycles of denaturation at 95°C for 40 s, annealing at 44°C for 45 s and elongation at 72°C for 1 min, with a final elongation step at 72°C for 8 min. The PCR products were then directly and bi-directionally Sanger sequenced on an ABI 3031 automated sequencer (Applied Biosystems) using the same primers and following the manufacturer’s protocol. The raw sequence editing and the generation of contiguous sequences were accomplished using CodonCode Aligner (CodonCode Corporation). Multiple sequence alignments were automatically generated using Muscle v3.8.31 [39] as implemented in Seaview v.4.4.0 [40] and verified manually.

### DNA extraction, PCR, library preparation and Illumina sequencing of mixed samples

The total genomic DNA was extracted from the six mixed samples of oligochaete fragments using the Qiagen DNeasy Tissue Kit (Qiagen, Hilden, Germany) following the manufacturer’s protocol. The fragment of COI gene was PCR amplified from each DNA extract as above. Each mixture extract was PCR amplified in duplicate (12 PCR in total). The products of each PCR were then purified individually using High Pure PCR Purification Kit (Roche Diagnostics). 50 ng of purified PCR products were then appended with Illumina PE adapter sequences in order to obtain one functional sequencing library per PCR replicate. This was performed using the TruSeq DNA Nano kit (Illumina) and following the kit instructions to include a unique index as a label for each library. The 12 libraries were then pooled in equimolar quantities prior to Illumina sequencing. The pool was sequenced on one MiSeq paired-end sequencing run of 2x251 cycles using a MiSeq Reagent Nano Kit v2 (Illumina).

### Analysis of sequences

The length of the sequencing reads was too short for the paired-end reads to overlap over the entire sequence of COI fragment (658 base pairs). Therefore, the processing of the fastq files was performed independently for the forward (R1) and reverse (R2) reads, and for each sample. First, the raw reads were quality filtered, so that only the sequences having an average quality of 30 or more and no more than one base with a quality below 30 were kept. Moreover, only the sequenced amplicons for which both the R1 and R2 paired reads had a high quality were kept. Then, the primer sequences were searched in these R1 and R2 reads allowing at most one difference (Levensthein’s edit distance, [41]). Again, the reads R1 and R2 were paired during this analysis. Indeed, the HCO (or LCO) primer sequence was first searched in the R1 read. If present, the corresponding LCO (or HCO) primer sequence was then searched in the paired R2 read. If none or only one of the primers could be found, both reads were discarded. At this point, we obtained two dataset, i.e. one for each end of the COI fragment: the HCO and the LCO datasets.

For each sample, two phylogenetic trees (one for LCO and another for HCO) comprising the sequences of oligochaete individuals (Sanger), the Illumina sequences and the sequences of our database [20] were constructed using the neighbour-joining method as implemented in Seaview v.4.4.0 [40], with 1,000 bootstrap replicates. Our COI database was augmented with four sequences belonging to *Marionina* sp, added during the present work (accession numbers: LN999380—LN999383). The vouchers (anterior parts of the specimens mounted between slide and coverslip in a permanent coating solution) corresponding to these sequences have been deposited at the Museum of Natural History of the city of Geneva. The Sanger sequences and the sequences of our database were trimmed to the maximum length of Illumina sequences before building trees. A 10% threshold of COI divergence was applied to segregate between species [20]. The intra- and inter-OTU distances were calculated using the K2P model in MEGA 5.1 [42]. The COI sequences that did not correspond to any sequence of our database were compared to Genbank (NCBI) sequences using BLAST (www.ncbi.nlm.nih.gov/BLAST/Blast.cgi) and to BOLD sequences (http://www.boldsystems.org/index.php/IDS_OpenIdEngine).

For each sample corresponding to a mix of oligochaete specimens, we had one dataset composed of OTUs delineated from Sanger sequencing and expressed in terms of number of specimens assigned to these OTUs, and four datasets composed of OTUs delineated from NGS sequences and expressed in terms of number of reads assigned to these OTUs. The four NGS datasets corresponded to 2 PCR replicates for the LCO end and 2 PCR replicates for the HCO end. Hence, we first built a single NGS dataset for each sample by averaging the number of reads per OTU found across the four datasets. Then, we compared the proportions of OTUs delineated from Sanger sequencing and NGS.

For some OTUs, we calculated the optimal correction factor that had to be applied to the NGS sequence abundances to minimize the difference between the expected proportions (Sanger-sequenced specimens) and the observed proportions (NGS reads) in the set of mixed samples. To do this, we multiplied the absolute NGS sequence abundance of an OTU in each sample by a factor ranging from 0.1 to 1 (steps of 0.05) and from 1 to 200 (steps of 1). Then, for each factor value, we re-calculated the resulting proportion of this OTU in terms of NGS sequences in the mixed samples. We examined for the OTU the average percent difference between the expected proportions and the observed proportions as a function of various factor values and selected the correction factor value that corresponded to the minimum average difference between the expected and observed proportions.

Linear regressions and Pearson’s test were performed to analyse the relationships between the proportion of Sanger sequenced OTUs and the proportion of OTUs in NGS data (per sample and for all samples combined). These relationships were also studied after application of the OTU-specific correction factors described previously. These analyses were performed using the Free Statistics and Forecasting Software [43].

### Biological quality of sediments: IOBS Oligochaete index

The IOBS is expressed as 10 times the total number of taxa present in a sample, divided by the percentage of the tubificids (former family Tubificidae, comprising among others the subfamilies Tubificinae and Rhyacodrilinae) with or without hair setae, whichever is dominant in the sediment sample [8, 9]. The index ranks the biological quality of sediments in five discrete classes: IOBS ≥ 6: very good; 6 > IOBS ≥ 3: good; 3 > IOBS ≥ 2: medium; 2 > IOBS ≥ 1: poor; IOBS < 1: bad. We calculated this index for each sample, with Sanger and NGS data and after correction of the sequence abundance data with the OTU-specific factors described previously.

## Results

### Sanger-sequenced specimen data

The PCR amplification and Sanger sequencing was successful for a large majority of specimens. Only 14 specimens out of a total of 330 could not be sequenced due to failed PCR amplification. The phylogenetic analyses of the 316 sequences allowed the identification of 31 OTUs. The number of OTUs per sample ranged from 10 to 15 (Table 1, S1 and S2 Tables).

**Table 1:** Numbers of specimens, of sequenced specimens and of OTUs (Sanger, NGS and total) per sample.

	S1	S2	S3	S4	S5	S6
Total number of specimens	43	42	28	78	32	107
Number of sequenced specimens (Sanger)	41	42	24	75	32	102
Number of OTUs with Sanger	15	12	10	12	10	14
Number of OTUs with NGS	14	12	12	13	10	17
Total number of OTUs (Sanger + NGS)	15	13	12	13	12	17

The 31 OTUs comprised 16 described morphospecies, 2 unidentified Enchytraeidae, 10 unidentified Tubificinae (including 4 with hair setae and 6 without hair setae), 2 *Marionina* sp. and one specimen that could not be assign with confidence to any family (indet 1). Among the morphospecies, 8 were cryptic (3 in *Tubifex tubifex* Müller 1774 and 5 in *Limnodrilus hoffmeisteri* Claparède 1862). 10 OTUs were totally new. They did not correspond to any sequence in Genbank and BOLD and matched no sequence in our COI database [20]. These new OTUs included 2 *Marionina* sp., 2 OTUs branching within Enchytraeidae, 2 within Tubificinae with hair setae and 3 within Tubificinae without hair setae.

### NGS sequence data

We obtained a total of 708,866 forward and reverse Illumina reads distributed evenly across the 12 sample libraries, with a minimum and maximum number of sequences per sample of 51,160 and 75,030, respectively. The quality filtering resulted into the removal of 56.6% and 86.6% of the R1 and R2 reads, respectively. Only 75,911 sequences were of high quality for both reads, and both the HCO and LCO primer sequences could be found in 64.3% of them (48,871 sequences). Interestingly, the filtered sequences were evenly distributed across replicates, and the numbers of unique sequences between the LCO and HCO versions of each sample were very similar, with an average difference of 9.6 unique sequences only (S3 Table). Most of the lineages that have been delineated from the full-length COI sequences could be identified based on the shorter NGS sequences (29 out of 31), i.e. based on the LCO and HCO ends analysed separately. The occurrence of a given OTU in a sample was detected in 6 cases based on the LCO sequence and not the HCO sequence, or vice versa. In 11 cases, the OTU could be identified in only one of the two PCR replicates.

A total of 33 OTUs were identified in NGS data. The number of OTUs per sample ranged from 10 to 17 (Table 1, S1 and S2 Tables). We identified 16 morphospecies, 2 Enchytraeidae sp., 2 *Marionina* sp., 12 unidentified Tubificinae, including 4 with hair setae and 8 without hair setae, and one specimen could not be assigned with confidence to any oligochaete family (indet 2). Among the morphospecies, 8 were cryptic (3 in *Tubifex tubifex* and 5 in *Limnodrilus hoffmeisteri*). 12 OTUs were totally new, including 2 *Marionina* sp., 2 OTUs branching with Enchytraeidae sp., 2 with Tubificinae with hair setae and 5 with Tubificinae without hair setae.

### Detection of OTUs

**Fig 1:**
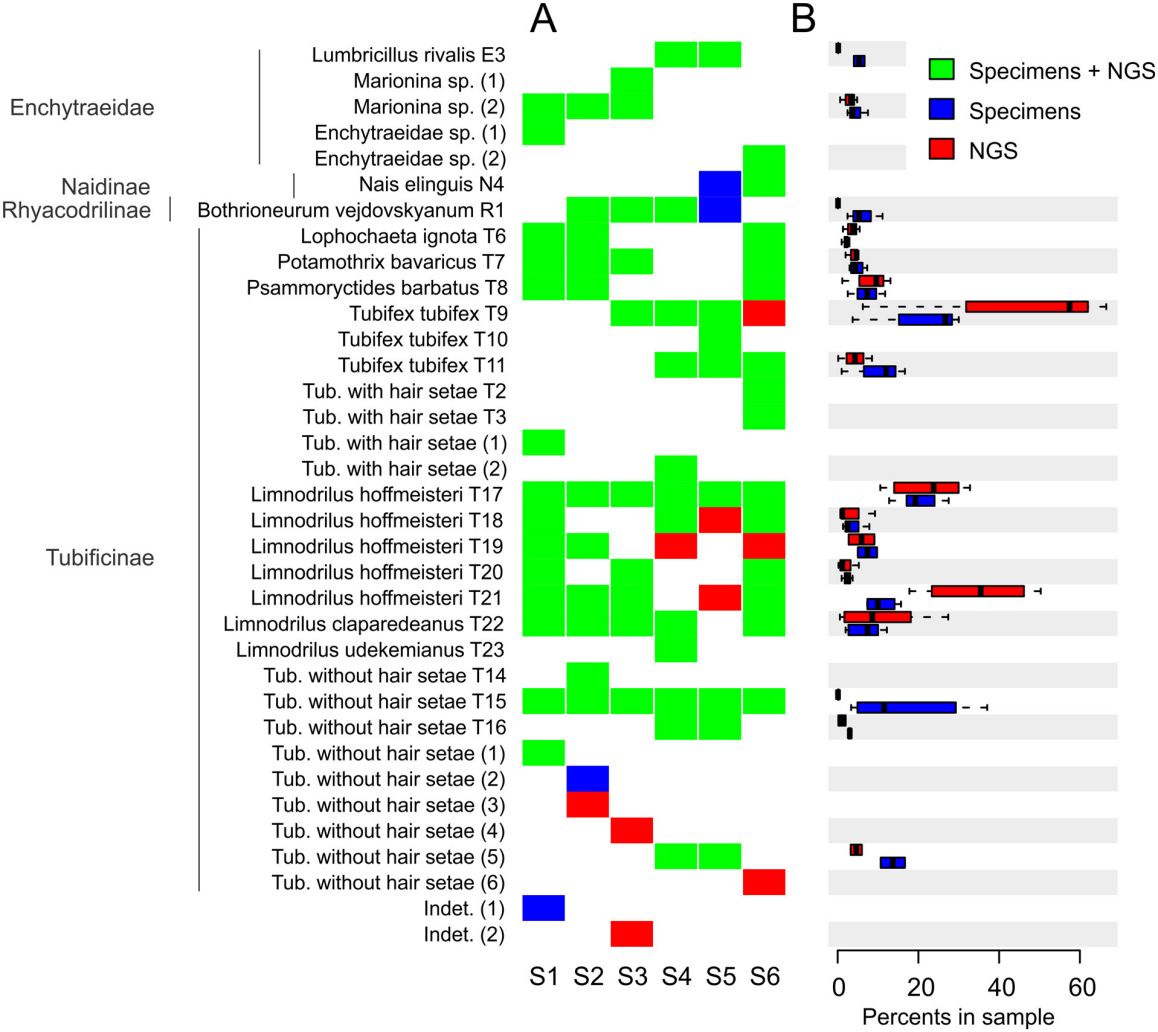
Detection of each OTU in the mixed samples by NGS. A. The heatmap indicates if a taxon present in a mixed sample could be detected in the NGS data (green) or not (blue). The OTUs present in the NGS data of a given sample but not identified by Sanger sequencing are indicated in red. B. The boxplots display the proportions of specimen sequences (blue) and of NGS sequences (red) for each taxon sequenced in at least two mixed samples. OTUs designated by a letter followed by a number are known OTUs [20] and OTUs designated by a number in brackets are new; Indet = unidentified.

Almost all of the OTUs (29 out of 31) that were Sanger sequenced could be identified in NGS data, based on the LCO and HCO fragments analysed separately. The placement of the NGS sequences in the phylogenetic framework allowed the discovery of four OTUs not inferred from the Sanger sequences (Fig 1). Three of them corresponded to unknown Tubificinae without hair setae and one could not be assigned with confidence to any oligochaete family (indet. 2). All these sequences were represented by few reads (≤ 0.031%). Moreover, there were five cases, in which the OTU was found in NGS but not in Sanger data for a given sample. Conversely, four OTUs (Tubificinae without hair setae (2), Indet 1, *Nais elinguis* Müller 1774 and *Bothrioneurum vejdovskyanum* Stolc 1886) identified by Sanger sequencing were not detected using NGS (Fig 1). All of them were represented by a single specimen.

### Relative abundance of OTUs

The proportions of NGS sequences did not reflect exactly the proportions of specimens, but the differences varied depending on the taxon (S1 and S2 Tables, S1 Fig). We identified eight OTUs for which the difference was important (ratios between the proportions of OTUs obtained with Sanger-sequenced specimen data and of OTUs obtained with NGS approach, or inversely, superior to 2 in at least two samples): *Limnodrilus hoffmeisteri* T21, *Tubifex tubifex* T9, *Bothrioneurum vejdovskyanum* R1, *Limnodrilus hoffmeisteri* T20, *Tubifex tubifex* T11, *Lumbricillus rivalis* Levinsen 1884 (OTU E3) as well as two Tubificinae without hair setae (T15 and indet 5) (S4 Table). The proportion of the two first taxa was overestimated by NGS analysis, while it was underestimated for the other OTUs. Interestingly, for all these OTUs except *Limnodrilus hoffmeisteri* T20, the difference in the relative abundance of specimens and sequences was consistent in terms of direction and magnitude in all the samples in which they were present (S4 Table). We applied optimal correction factor values that reduced the skew in all samples for OTUs that were present in at least four samples (S1 and S2 Figs): *Limnodrilus hoffmeisteri* T21, *Tubifex tubifex* T9, Tubificinae without hair setae T15 and *Bothrioneurum vejdovskyanum* R1. Their factors were 0.19 (resulting minimum difference: 1.11%), 0.22 (min. diff. 2.15%), 149 (min. diff. 3.43%) and 132 (min. diff. 1.46%), respectively.

**Fig 2:**
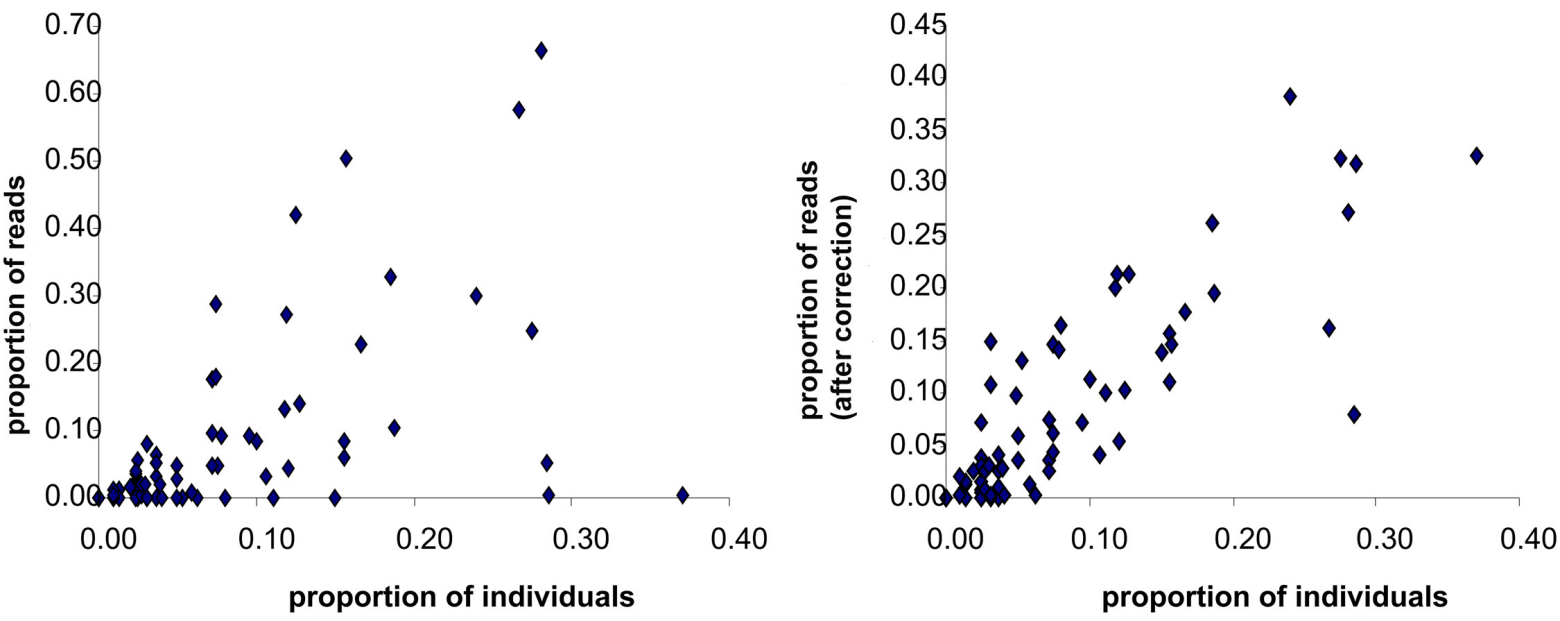
Relationships between the proportions of OTUs obtained with Sanger-sequenced specimen data and NGS approach without correction of sequence abundances (left) and after correction (right) per taxon and per sample, for all samples (1–6).

The correlations between expected OTU proportions (Sanger) and NGS sequence proportions were significant in samples 1, 4, 5 and 6 but not significant in samples 2 and 3 (Table 2, Fig 2). The absence of correlation in these samples could be due to the underestimation of OTU T15 percentage in NGS data. The correlation between proportion of specimens and NGS sequences in the six samples combined was significant (Table 2, Fig 2). When the numbers of sequences of the taxa Tubificinae without hair setae T15, *Bothrioneurum vejdovskyanum* R1, *Limnodrilus hoffmeisteri* T21 and *Tubifex tubifex* T9 were corrected by applying their respective correction factors (see above), the correlations between proportions were very significant in each sample and in the combined samples (Table 2, Fig 2).

**Table 2:** R and P values (Pearson test) of the relationships between the OTU proportions obtained with Sanger-sequenced specimen data and with NGS approach per sample (n = 12–17) and for all samples (n = 82).

		S1	S2	S3	S4	S5	S6	S1-S6
% Ind—% seqs	R	0.627	0.318	0.154	0.892	0.803	0.520	0.585
	P	0.0123	NS	NS	4.09*10^−5^	0.0016	0.0326	6.301*10^−9^
% Ind—Corr % seqs	R	0.968	0.945	0.935	0.788	0.851	0.635	0.852
	P	3.2*10^−9^	1.1*10^−6^	7.8*10^−6^	0.00138	0.00044	0.00614	<10^−10^

% Ind = percentages of OTUs obtained with Sanger-sequenced specimen data. % seqs = percentages of OTUs obtained with NGS approach without correction of sequence abundances. Corr % seqs = percentages of OTUs obtained with NGS approach after correction of sequence abundances. NS = not significant

### IOBS Oligochaete Index calculation

The IOBS values inferred from the analysis of the specimens and NGS data were similar in samples 1, 2, 3 but different in samples 4, 5 and 6 (Table 3). The underestimation in NGS of OTU T15 did not have an influence on IOBS result in samples 1–3. In samples 5 and 6, the difference between Sanger and NGS data was explained by the overestimation in NGS of OTUs T9 (sample 5) and T21 (sample 6). In sample 4, the difference was explained by the overestimation of OTU T9 and the underestimation of OTUs R1 and T15. After application of the correction factors to the sequence abundances (Tubificinae without hair setae T15, *Bothrioneurum vejdovskyanum* R1, *Limnodrilus hoffmeisteri* T21 and *Tubifex tubifex* T9), the IOBS values of sample 4, 5 and 6 approached the reference value but remained too low, indicating poor rather than medium quality in samples 4 and 5 and medium rather than good quality of sediments in sample 6 (Table 3). These differences were explained among others by the overestimation of OTU T17 in sample 4, the overestimation of OTU T10 in sample 5 and the underestimation of OTU N4 in sample 6 (S2 Table, S1 Fig). These three OTUs could not be corrected, as OTUs T10 and N4 were present in only one sample and OTU T17 was not overestimated in all samples.

**Table 3:** Number of OTUs, percentage of tubificids with or without hair setae and IOBS values of each sample obtained with Sanger-sequenced specimen data and NGS approach with uncorrected and corrected sequence abundances.

		S1	S2	S3	S4	S5	S6
Sanger seq	Number of OTUs	15	12	10	12	10	14
	% of tubificids	77.5	80.95	85.19	56	46.88	44.12
	IOBS	**1.94**	**1.48**	**1.17**	*2*.*14*	*2*.*13*	3.17
NGS, uncorr seq abund	Number of OTUs	14	12	12	13	10	17
	% of tubificids	80.47	81.26	85.4	37.07	83.04	75.45
	IOBS	**1.74**	**1.48**	**1.41**	3.5	**1.2**	*2*.*25*
NGS, corr seq abund	Number of OTUs	14	12	12	13	10	17
	% of tubificids	74.53	85.41	92.17	76.84	57.88	62.77
	IOBS	**1.88**	**1.40**	**1.3**	**1.69**	**1.73**	*2*.*71*

Sanger seq = Sanger sequencing; uncorr seq abund = uncorrected sequence abundances; corr seq abund = corrected sequence abundances. IOBS values in bold: poor biological quality; IOBS values in italics: medium quality; IOBS values underlined: good quality

## Discussion

Our results show that the NGS approach successfully detects the vast majority of oligochaete OTUs present in the mixed samples. This confirms the excellent detection ability of the NGS approach, as demonstrated by previous studies of aquatic insects [23, 33, 35]. There were only four cases, for which the NGS failed to detect an OTU, but in all cases a single specimen represented the missing OTU. The absence of these OTUs in NGS can be explained either by the very low quantity of DNA corresponding to these specimens or by amplification bias due to weak affinities of the PCR primers and to competition among species for PCR primer binding.

In our data, we reported nine cases with OTUs occurring in the NGS data exclusively, but all related to extremely rare sequences. Some of the unexpected OTUs detected with NGS could correspond to specimens for which the PCR amplification failed and therefore they could not be Sanger-sequenced (in samples 3, 4 and 6). Among other factors that can explain these discrepancies, there is a general tendency of NGS to generate additional sequence diversity, and thus to reveal the presence of species that were not expected in the samples [44, 45]. The presence of unexpected OTUs could be also explained by the result of cross-contamination events, which are difficult to avoid in multiplexed designs [46] and more generally given the huge sample coverage of NGS [47]. Alternatively, OTUs that occurred only in NGS could represent rare divergent copies of COI gene (pseudogenes or nuclear mitochondrial DNA). Such copies are frequently reported in other invertebrates [48, 49] and it is probable that they are present also in oligochaetes.

While the performance of NGS approach to detect species presence was indisputable, the quantitative interpretation of NGS data was more problematic. It is generally assumed that the abundance of specimens is not directly related to the abundance of sequences due to various biases [50]. Biological biases such as the gene copy number and biomass variations may be coupled to technical biases, including differential PCR primer hybridization affinities to different template molecules and amplification efficiencies [51, 52].

As the relative abundance of some species was skewed consistently in our samples (over- or under-estimation) and at comparable magnitudes, this suggests that the degree of affinity of the PCR primers and the competition among the diverse template DNA sequences for the PCR primers play a dominant role in observed abundance variation. PCR amplification efficiencies rather than biomass variations could explain these results as it is unlikely that the tissue fragments added in different mixtures were repeatedly larger or smaller for all the specimens of the same OTUs.

To overcome the sequence abundance issue, we empirically defined correction factors based on the difference between the expected and observed relative abundances across samples of four OTUs. For each OTU, we searched for correction factors that reduced this skew to a minimum and found that a unique factor averaged over all samples also performed efficiently to reduce the skew when applied for each sample separately. The application of such factors allowed a more accurate estimation of the relative abundances of each oligochaete community. Assuming that the COI sequences of different oligochaete species cannot be amplified with the same efficiencies but within specific magnitudes, we propose that it is possible to exploit the sequence abundance data provided that they could be corrected. As a result, the sequence abundance data could better relate to the specimen abundance data, which is essential for robust NGS-based monitoring studies.

Of course, such empirically defined correction factors should be tested and validated on more data. It would be important to evaluate the amplification rate of these four OTUs when oligochaete communities change and when other taxocenoses than oligochaetes are present. Furthermore, the degree of PCR primer affinity to these OTUs could be assessed by performing Q-PCR method.

The discrepancies between the expected OTU abundances (Sanger) and NGS sequence relative abundances are not new: Hajibabaei et al. [23] and Carew et al. [33] also missed some species present in low abundances, observed the over- or under-estimation of some species and found unexpected sequences in their NGS data, that they ascribed to 454 pyrosequencing errors or contaminants. It is also known that the filtering of the sequence data might discard rare species, while keeping abundant errors [36]. Moreover, the presence of sequence artefacts in the samples could contribute to the slight variations in the relative sequence abundances, such as those observed for the OTUs affected by weak skews. Indeed, it cannot be excluded that our analyses were affected by the presence of sequence chimeras since the detection of such events was difficult based on incomplete sequences (i.e. on the LCO and HCO ends of the fragment of COI gene). Yet, the LCO and HCO datasets provided similar results on the diversity and relative abundance for each sample, as well as the PCR replicates. As a few OTUs could be detected with the LCO sequence but not with the HCO sequence and vice versa, and in one replicate but not in the other replicate, we recommend using values averaged over LCO, HCO and at least two replicates for each sample. There is not doubt that this constraint may be alleviated by future improvement towards longer read lengths.

Bringing solution to these technical issues is essential from the point of view of practical application of NGS approach to the evaluation of ecosystem quality through the calculation of the IOBS index. We observed that in three samples, the differences of proportion of some OTUs had a significant influence on IOBS results. In these samples, the application of the correction factors allowed us to make the IOBS result inferred from NGS quite close to the expected result.

As tubificids (former Tubificidae) with and without hair setae do not constitute monophyletic groups [20], the application of the NGS-based IOBS index requires having a comprehensive COI reference database of the tubificids, which is not easily realizable for different geographical regions. However, it is not necessary that all OTUs of the reference database are assigned to a species name, a determination whether they possess or not hair setae is sufficient. This issue could be overcome by modifying the IOBS index formula, for example the total number of genetic OTUs could be divided by the percentage of tubificid OTUs (with and without hair setae). But any modification of the index should be validated against physicochemical data.

In addition to the issues mentioned above, another parameter that should be taken into consideration is the underestimation of the species richness by morphological approach. As shown by Vivien et al. [20], the morphological identification underestimates the diversity of aquatic oligochaetes for two reasons: (1) a large proportion of specimens is immature and cannot be identified, (2) some common morphospecies comprise a high level of cryptic diversity. The fact that the IOBS values based on genetic analyses are always higher than IOBS results based on morphological analyses may lead to an overestimation of the quality of sediments. This issue could be overcome by (1) modifying the IOBS formula, (2) modifying the quality classes or (3) combining the different OTUs of a species (cryptic species) in one species. However, the last solution is not ideal because the cryptic species can show differences in resistance to pollution and their distinction can be important for ecological studies and may help interpreting environmental conditions [20].

To conclude, this experimental study confirms the potential of NGS to identify species in the community of oligochaetes. It also shows that it is possible to infer the species abundance on the condition of using robust species-specific correction factors. These promising results open new perspectives for the routine use of NGS-based analysis of oligochaete diversity not only from the sorted specimen mixtures but also directly from the sedimentary DNA samples. This would greatly contribute to wider application of oligochaete-based indices for the assessment of biological quality of freshwater ecosystems.

## Supporting Information

S1 Fig OTU percentages obtained with Sanger-sequenced specimen data (Ind) and NGS approach without correction of sequence abundances (reads) and after correction of sequence abundances (corr reads). OTUs designated by a letter followed by a number are known OTUs [20]; OTUs designated by a number in brackets are new. Indet = unidentified.
(PDF)

S2 Fig Absolute differences between the proportions of specimens (Sanger sequencing) and of NGS sequences for 12 OTUs as a function of various correction factor values. For each OTU is shown the average difference calculated over 2 to 6 mixed samples (y axis) and the values of the corresponding correction factors (x axis). The minimum difference values are indicated by symbols. The taxa are separated in three panels because of the variation in magnitude of the correction factors that must be applied to minimize the difference.
(PDF)

S1 Table Data per sample (samples 1–3). Number of specimens per OTU (Ind), percentages of OTUs obtained with Sanger-sequenced specimen data (% Ind), read means (NGS), percentages of read means (% reads), corrected read means (corr read means) and percentages of corrected read means (% corr reads).
(DOC)

S2 Table Data per sample (samples 4–6). Number of specimens per OTU (Ind), percentages of OTUs obtained with Sanger-sequenced specimen data (% Ind), read means (NGS), percentages of read means (% reads), corrected read means (corr read means) and percentages of corrected read means (% corr reads).
(DOC)

S3 Table High-throughput sequencing data. For each sequenced PCR replicate (Library) of each of the six mixed samples (Sample) are indicated the numbers of sequences in total (Total reads), as well as after quality filtering of the R1 and R2 reads (Quality), after selection of the pairs remaining in both R1 and R2 (Shared) and for which the primer sequences could be found (Primer). The number of unique sequences and OTUs corresponding to these sequences after dereplication and clustering are indicated for each end of the sequenced amplicons (LCO and HCO), in the columns “Unique sequences” and “OTUs” respectively.
(DOC)

S4 Table Ratios between the proportions of OTUs obtained with Sanger-sequenced specimen data and of OTUs obtained with NGS approach for each sample and OTU. (DOC)
