# Supplementary figures and images for: Next-Generation Sequencing of Aquatic Oligochaetes: Comparison of Experimental Communities
Source: PLoS One. 2016 Feb 11;11(2):e0148644. doi: 10.1371/journal.pone.0148644 (PMC4750909; doi:10.1371/journal.pone.0148644)

sample 1

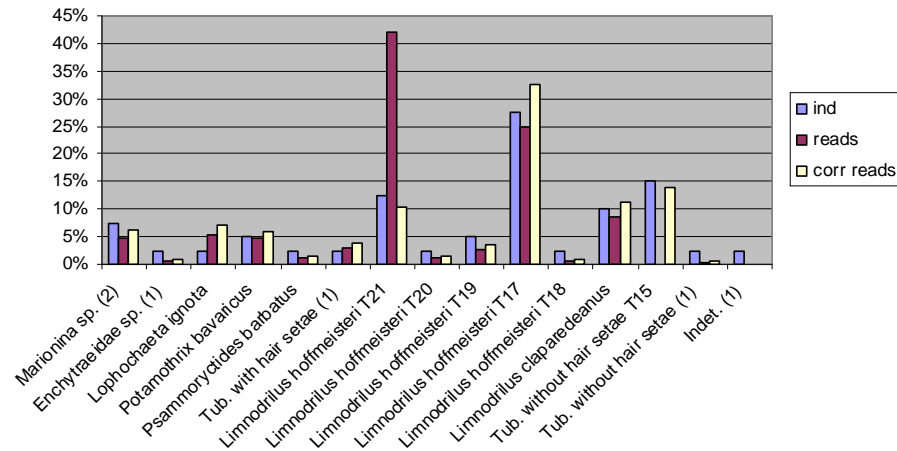

sample 2

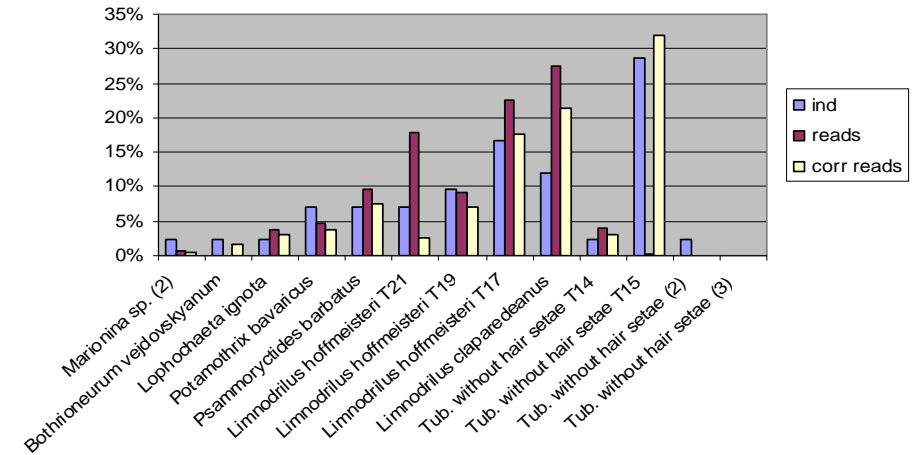

sample 3

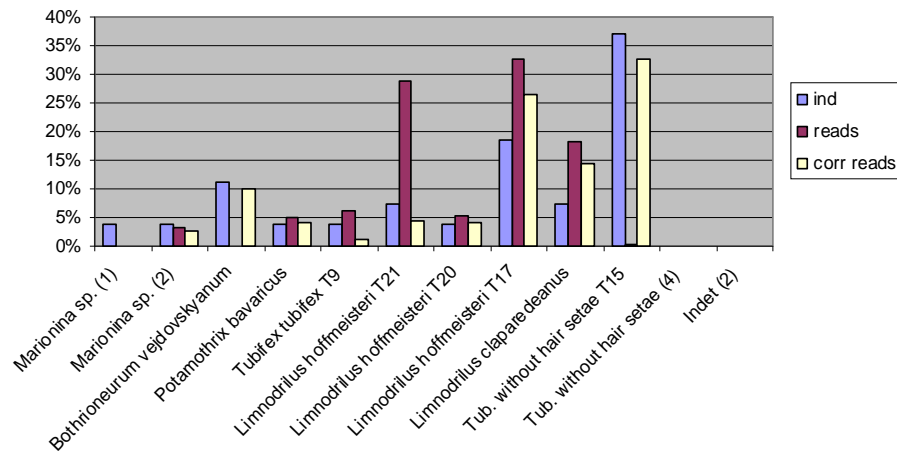

sample 4

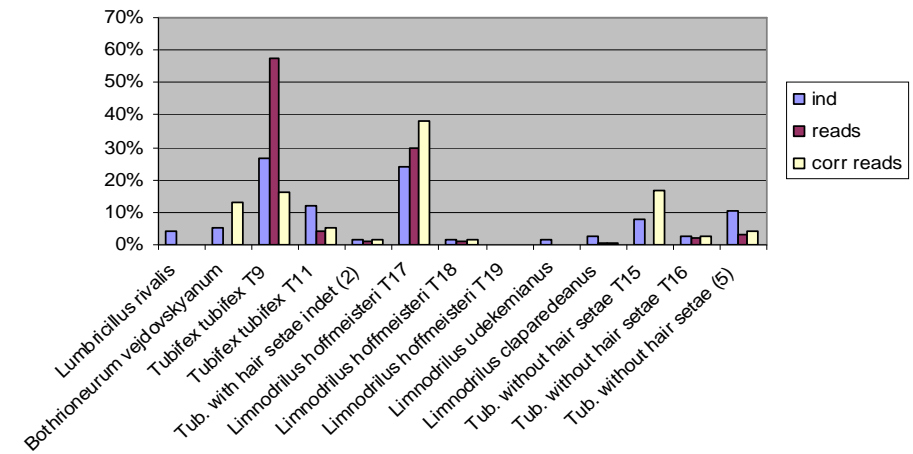

sample 5

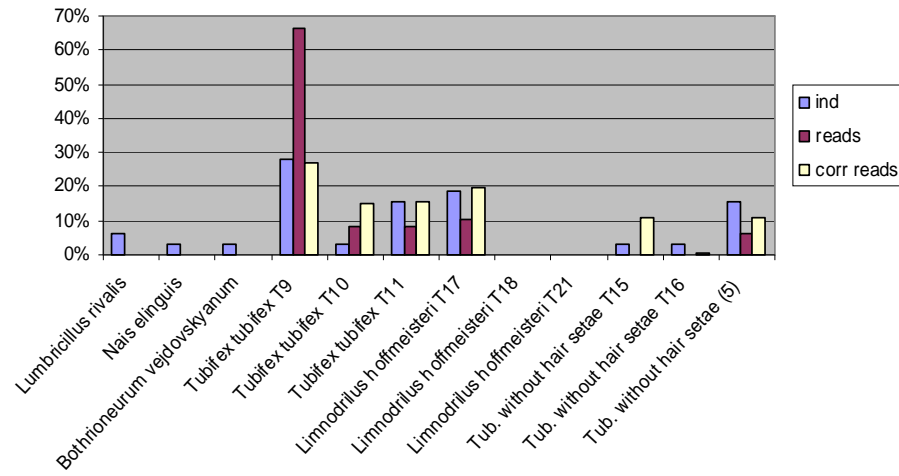

sample 6

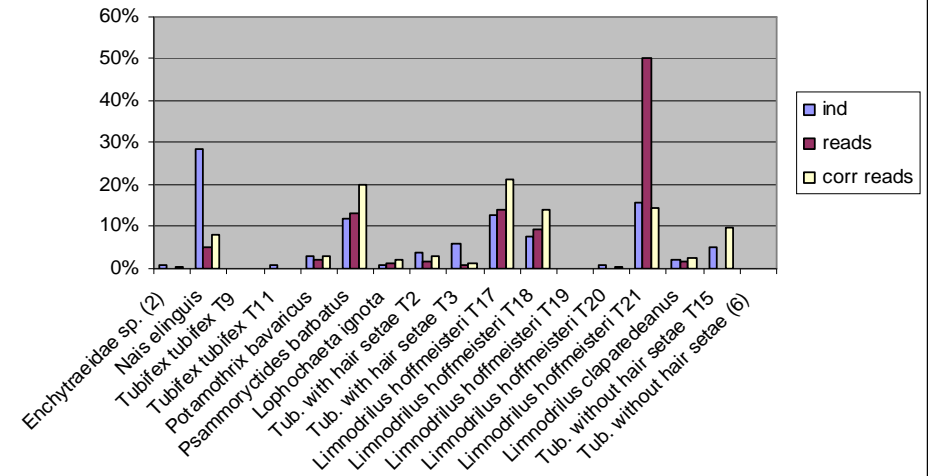

Supplement: S1 Fig — OTUs designated by a letter followed by a number are known OTUs [20]; OTUs designated by a number in brackets are new. Indet = unidentified. (PDF) [file pone.0148644.s001.pdf]
