# Supplementary material for: Next-Generation Sequencing of Aquatic Oligochaetes: Comparison of Experimental Communities
Source: PLoS One. 2016 Feb 11;11(2):e0148644. doi: 10.1371/journal.pone.0148644 (PMC4750909; doi:10.1371/journal.pone.0148644)
Supplement: S2 Fig — For each OTU is shown the average difference calculated over 2 to 6 mixed samples (y axis) and the values of the corresponding correction factors (x axis). The minimum difference values are indicated by symbols. The taxa are separated in three panels because of the variation in magnitude of the correction factors that must be applied to minimize the difference. (PDF) [file pone.0148644.s002.pdf]

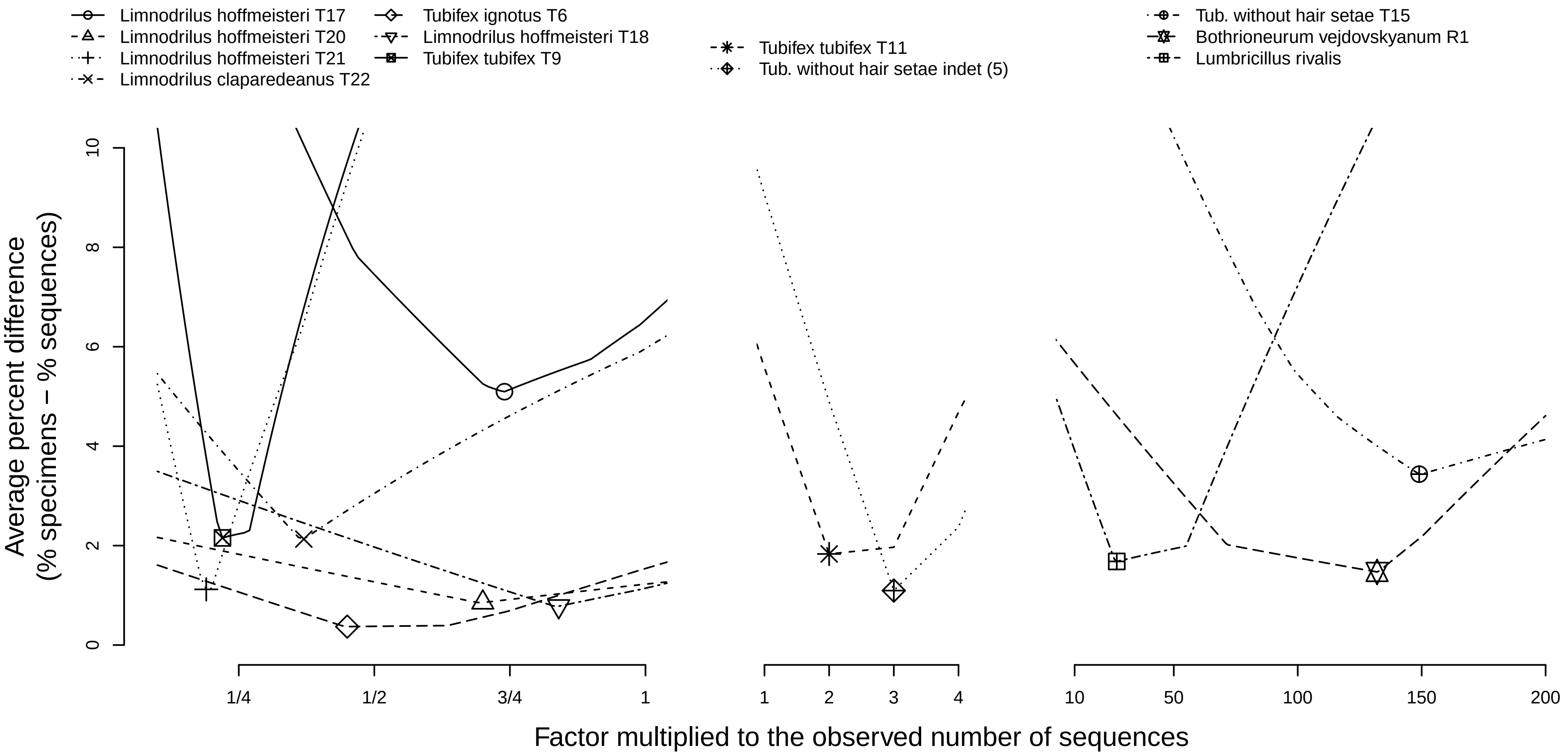
Limnodrilus hoffmeisteri T17
Limnodrilus hoffmeisteri T20
Limnodrilus hoffmeisteri T21
Limnodrilus claparedeanus T22
Tubifex ignotus T6
Limnodrilus hoffmeisteri T18
Tubifex tubifex T9
Tubifex tubifex T11
Tub. without hair setae indet (5)
Tub. without hair setae T15
Bothrioneurum vejdovskyanum R1
Lumbricillus rivalis
Average percent difference
(% specimens – % sequences)
0
2
4
6
8
10
1/4
1/2
3/4
1
1
2
3
4
10
50
100
150
200
Factor multiplied to the observed number of sequences
