# Supplementary material for: Next-Generation Sequencing of Aquatic Oligochaetes: Comparison of Experimental Communities
Source: PLoS One. 2016 Feb 11;11(2):e0148644. doi: 10.1371/journal.pone.0148644 (PMC4750909; doi:10.1371/journal.pone.0148644)
Supplement: S1 Table — Number of specimens per OTU (Ind), percentages of OTUs obtained with Sanger-sequenced specimen data (% Ind), read means (NGS), percentages of read means (% reads), corrected read means (corr read means) and percentages of corrected read means (% corr reads). (DOC) [file pone.0148644.s003.doc]

|  | **Sample 1** | | | | | | **Sample 2** | | | | | | **Sample 3** | | | | | |
| --- | --- | --- | --- | --- | --- | --- | --- | --- | --- | --- | --- | --- | --- | --- | --- | --- | --- | --- |
|  | Ind | % ind | read means | % reads | corr read means | % corr reads | Ind | % ind | read means | % reads | corr read means | % corr reads | Ind | % ind | read means | % reads | corr read means | % corr reads |
| Marionina sp. (1) |  |  |  |  |  |  |  |  |  |  |  |  | 1 | 3.70 | 1.3 | 0.02 | 1.3 | 0.019 |
| Marionina sp. (2) | 3 | 7.5 | 184.3 | 4.83 | 184.3 | 6.30 | 1 | 2.38 | 29.5 | 0.61 | 29.5 | 0.48 | 1 | 3.70 | 176.3 | 3.33 | 176.3 | 2.67 |
| Enchytraeidae sp. (1) | 1 | 2.5 | 22.3 | 0.58 | 22.3 | 0.76 |  |  |  |  |  |  |  |  |  |  |  |  |
| *Bothrioneurum vejdovskyanum* R1 |  |  |  |  |  |  | 1 | 2.38 | 0.8 | 0.02 | 98.3 | 1.59 | 3 | 11.11 | 5.0 | 0.09 | 655.0 | 9.92 |
| *Lophochaeta ignota* T6 | 1 | 2.5 | 207.8 | 5.44 | 207.8 | 7.10 | 1 | 2.38 | 182.3 | 3.79 | 182.3 | 2.95 |  |  |  |  |  |  |
| *Potamothrix bavaricus* T7 | 2 | 5.0 | 176.5 | 4.62 | 176.5 | 6.03 | 3 | 7.14 | 229.0 | 4.76 | 229.0 | 3.71 | 1 | 3.70 | 266.5 | 5.03 | 266.5 | 4.04 |
| *Psammoryctides barbatus* T8 | 1 | 2.5 | 43.5 | 1.14 | 43.5 | 1.49 | 3 | 7.14 | 460.8 | 9.58 | 460.8 | 7.46 |  |  |  |  |  |  |
| *Tubifex tubifex* T9 |  |  |  |  |  |  |  |  |  |  |  |  | 1 | 3.70 | 329.8 | 6.22 | 72.5 | 1.10 |
| Tub. with hair setae (1) | 1 | 2.5 | 111.0 | 2.91 | 111.0 | 3.79 |  |  |  |  |  |  |  |  |  |  |  |  |
| *Limnodrilus hoffmeisteri* T21 | 5 | 12.5 | 1602.3 | 41.98 | 304.4 | 10.41 | 3 | 7.14 | 854.5 | 17.76 | 162.4 | 2.63 | 2 | 7.41 | 1530.8 | 28.88 | 290.8 | 4.41 |
| *Limnodrilus hoffmeisteri* T20 | 1 | 2.5 | 43.8 | 1.15 | 43.8 | 1.50 |  |  |  |  |  |  | 1 | 3.70 | 278.0 | 5.24 | 278.0 | 4.21 |
| *Limnodrilus hoffmeisteri* T19 | 2 | 5.0 | 104.5 | 2.74 | 104.5 | 3.57 | 4 | 9.52 | 437.5 | 9.09 | 437.5 | 7.08 |  |  |  |  |  |  |
| *Limnodrilus hoffmeisteri* T17 | 11 | 27.5 | 949.3 | 24.87 | 949.3 | 32.45 | 7 | 16.67 | 1089.5 | 22.65 | 1089.5 | 17.64 | 5 | 18.52 | 1737.5 | 32.78 | 1737.5 | 26.33 |
| *Limnodrilus hoffmeisteri* T18 | 1 | 2.5 | 25.8 | 0.67 | 25.8 | 0.88 |  |  |  |  |  |  |  |  |  |  |  |  |
| *Limnodrilus claparedeanus* T22 | 4 | 10.0 | 327.3 | 8.58 | 327.3 | 11.19 | 5 | 11.90 | 1317.3 | 27.38 | 1317.3 | 21.32 | 2 | 7.41 | 960.8 | 18.12 | 960.8 | 14.56 |
| Tub. without hair setae T14 |  |  |  |  |  |  | 1 | 2.38 | 195.3 | 4.06 | 195.3 | 3.16 |  |  |  |  |  |  |
| Tub. without hair setae T15 | 6 | 15.0 | 2.8 | 0.07 | 409.8 | 14.01 | 12 | 28.57 | 13.3 | 0.28 | 1974.3 | 31.96 | 10 | 37.04 | 14.5 | 0.27 | 2160.5 | 32.74 |
| Tub. without hair setae (1) | 1 | 2.5 | 15.5 | 0.41 | 15.5 | 0.53 |  |  |  |  |  |  |  |  |  |  |  |  |
| Tub. without hair setae (2) |  |  |  |  |  |  | 1 | 2.38 | 0.0 | 0.000 | 0.0 | 0.00 |  |  |  |  |  |  |
| Indet. (1) | 1 | 2.5 | 0.0 | 0.00 | 0.0 | 0.00 |  |  |  |  |  |  |  |  |  |  |  |  |
| Tub. without hair setae (3) |  |  |  |  |  |  | 0 | 0.00 | 1.5 | 0.031 | 1.5 | 0.02 |  |  |  |  |  |  |
| Tub. without hair setae (4) |  |  |  |  |  |  |  |  |  |  |  |  | 0 | 0.00 | 0.3 | 0.005 | 0.25 | 0.004 |
| Indet. (2) |  |  |  |  |  |  |  |  |  |  |  |  | 0 | 0.00 | 0.3 | 0.005 | 0.25 | 0.004 |

Lineages designated by a letter followed by a number are known lineages [20]

Lineages designated by a number in brackets are new

Indet. = unidentified
