# Supplementary material for: Next-Generation Sequencing of Aquatic Oligochaetes: Comparison of Experimental Communities
Source: PLoS One. 2016 Feb 11;11(2):e0148644. doi: 10.1371/journal.pone.0148644 (PMC4750909; doi:10.1371/journal.pone.0148644)
Supplement: S2 Table — Number of specimens per OTU (Ind), percentages of OTUs obtained with Sanger-sequenced specimen data (% Ind), read means (NGS), percentages of read means (% reads), corrected read means (corr read means) and percentages of corrected read means (% corr reads). (DOC) [file pone.0148644.s004.doc]

|  | **Sample 4** | | | | | | **Sample 5** | | | | | | **Sample 6** | | | | | |
| --- | --- | --- | --- | --- | --- | --- | --- | --- | --- | --- | --- | --- | --- | --- | --- | --- | --- | --- |
|  | Ind | % ind | read means | % reads | corr read means | % corr reads | Ind | % ind | read means | % reads | corr read means | % corr reads | Ind | % ind | read means | % reads | corr read means | % corr reads |
| Enchytraeidae sp. (2) |  |  |  |  |  |  |  |  |  |  |  |  | 1 | 0.98 | 7.3 | 0.14 | 7.3 | 0.21 |
| *Lumbricillus rivalis* E3 | 3 | 4.00 | 5.0 | 0.16 | 5 | 0.20 | 2 | 6.25 | 2.5 | 0.13 | 2.5 | 0.24 |  |  |  |  |  |  |
| *Nais elinguis* N4 |  |  |  |  |  |  | 1 | 3.13 | 0 | 0.00 | 0.0 | 0.00 | 29 | 28.43 | 270.3 | 5.21 | 270.3 | 7.95 |
| *Bothrioneurum vejdovskyanum* R1 | 4 | 5.33 | 2.5 | 0.08 | 327.5 | 13.18 | 1 | 3.13 | 0 | 0.00 | 0.0 | 0.00 |  |  |  |  |  |  |
| *Tubifex tubifex* T9 | 20 | 26.67 | 1824.3 | 57.45 | 401.3 | 16.15 | 9 | 28.13 | 1281 | 66.49 | 281.8 | 27.16 | 0 | 0.00 | 1.3 | 0.02 | 0.28 | 0.01 |
| *Tubifex tubifex* T10 |  |  |  |  |  |  | 1 | 3.13 | 155 | 8.05 | 155.0 | 14.94 |  |  |  |  |  |  |
| *Tubifex tubifex* T11 | 9 | 12.00 | 135.8 | 4.27 | 135.8 | 5.46 | 5 | 15.63 | 163.8 | 8.50 | 163.8 | 15.78 | 1 | 0.98 | 7 | 0.13 | 7 | 0.21 |
| *Potamothrix bavaricus* T7 |  |  |  |  |  |  |  |  |  |  |  |  | 3 | 2.94 | 101.5 | 1.96 | 101.5 | 2.99 |
| *Psammoryctides barbatus* T8 |  |  |  |  |  |  |  |  |  |  |  |  | 12 | 11.76 | 678.5 | 13.08 | 678.5 | 19.96 |
| *Lophochaeta ignota* T6 |  |  |  |  |  |  |  |  |  |  |  |  | 1 | 0.98 | 67.8 | 1.31 | 67.8 | 1.99 |
| Tub. with hair setae T2 |  |  |  |  |  |  |  |  |  |  |  |  | 4 | 3.92 | 98.8 | 1.90 | 98.8 | 2.90 |
| Tub. with hair setae T3 |  |  |  |  |  |  |  |  |  |  |  |  | 6 | 5.88 | 42 | 0.81 | 42 | 1.24 |
| Tub. with hair setae (2) | 1 | 1.33 | 33.3 | 1.05 | 33.3 | 1.34 |  |  |  |  |  |  |  |  |  |  |  |  |
| *Limnodrilus hoffmeisteri* T17 | 18 | 24.00 | 951.3 | 29.96 | 951.3 | 38.29 | 6 | 18.75 | 203.3 | 10.55 | 203.3 | 19.59 | 13 | 12.75 | 725.5 | 13.98 | 725.5 | 21.34 |
| *Limnodrilus hoffmeisteri* T18 | 1 | 1.33 | 36.3 | 1.14 | 36.3 | 1.46 | 0 | 0.00 | 0.5 | 0.026 | 0.5 | 0.05 | 8 | 7.84 | 479.5 | 9.24 | 479.5 | 14.10 |
| *Limnodrilus hoffmeisteri* T19 | 0 | 0.00 | 0.5 | 0.016 | 0.5 | 0.02 |  |  |  |  |  |  | 0 | 0.00 | 0.3 | 0.005 | 0.3 | 0.01 |
| *Limnodrilus hoffmeisteri* T20 |  |  |  |  |  |  |  |  |  |  |  |  | 1 | 0.98 | 10.5 | 0.20 | 10.5 | 0.31 |
| *Limnodrilus hoffmeisteri* T21 |  |  |  |  |  |  | 0 | 0.00 | 1 | 0.052 | 0.2 | 0.02 | 16 | 15.69 | 2609.3 | 50.29 | 495.8 | 14.58 |
| *Limnodrilus udekemianus* T23 | 1 | 1.33 | 1.5 | 0.05 | 1.5 | 0.06 |  |  |  |  |  |  |  |  |  |  |  |  |
| *Limnodrilus claparedeanus* T22 | 2 | 2.67 | 17.5 | 0.55 | 17.5 | 0.70 |  |  |  |  |  |  | 2 | 1.96 | 86.8 | 1.67 | 86.8 | 2.55 |
| Tub. without hair setae T15 | 6 | 8.00 | 2.8 | 0.09 | 409.8 | 16.49 | 1 | 3.13 | 0.8 | 0.04 | 111.8 | 10.77 | 5 | 4.90 | 2.3 | 0.043 | 335.3 | 9.86 |
| Tub. without hair setae T16 | 2 | 2.67 | 62.8 | 1.98 | 62.8 | 2.53 | 1 | 3.13 | 3.3 | 0.17 | 3.3 | 0.31 |  |  |  |  |  |  |
| Tub. without hair setae (5) | 8 | 10.67 | 102.3 | 3.22 | 102.3 | 4.12 | 5 | 15.63 | 115.5 | 6.00 | 115.5 | 11.13 |  |  |  |  |  |  |
| Tub. without hair setae (6) |  |  |  |  |  |  |  |  |  |  |  |  | 0 | 0.00 | 0.5 | 0.010 | 0.5 | 0.015 |

Lineages designated by a letter followed by a number are known lineages [20]

Lineages designated by a number in brackets are new
