# Supplementary material for: Next-Generation Sequencing of Aquatic Oligochaetes: Comparison of Experimental Communities
Source: PLoS One. 2016 Feb 11;11(2):e0148644. doi: 10.1371/journal.pone.0148644 (PMC4750909; doi:10.1371/journal.pone.0148644)
Supplement: S3 Table — For each sequenced PCR replicate (Library) of each of the six mixed samples (Sample) are indicated the numbers of sequences in total (Total reads), as well as after quality filtering of the R1 and R2 reads (Quality), after selection of the pairs remaining in both R1 and R2 (Shared) and for which the primer sequences could be found (Primer). The number of unique sequences and OTUs corresponding to these sequences after dereplication and clustering are indicated for each end of the sequenced amplicons (LCO and HCO), in the columns “Unique sequences” and “OTUs” respectively. (DOC) [file pone.0148644.s005.doc]

|  |  |  | Quality | |  |  | Unique sequences | | OTUs | |
| --- | --- | --- | --- | --- | --- | --- | --- | --- | --- | --- |
| Sample | Library | Total reads | R1 | R2 | Shared | Primer | LCO | HCO | LCO | HCO |
| 1 | 2 | 54206 | 23911 | 6069 | 4918 | 3181 | 429 | 442 | 22 | 23 |
|  | 4 | 61433 | 26812 | 8489 | 6786 | 4483 | 553 | 554 | 27 | 27 |
| 2 | 5 | 56610 | 25313 | 5309 | 4338 | 2516 | 440 | 445 | 31 | 30 |
|  | 6 | 65193 | 29019 | 12711 | 10299 | 7219 | 1035 | 1015 | 40 | 36 |
| 3 | 7 | 75032 | 33382 | 10800 | 8751 | 5804 | 758 | 735 | 36 | 35 |
|  | 12 | 60159 | 26340 | 9645 | 7692 | 4998 | 624 | 630 | 40 | 39 |
| 4 | 13 | 54208 | 22288 | 7389 | 5724 | 3483 | 502 | 503 | 18 | 17 |
|  | 14 | 51163 | 20720 | 6199 | 4760 | 2889 | 407 | 398 | 18 | 18 |
| 5 | 15 | 58573 | 23612 | 4809 | 3703 | 1926 | 317 | 306 | 13 | 13 |
|  | 16 | 52209 | 21897 | 4369 | 3503 | 1966 | 341 | 329 | 24 | 24 |
| 6 | 18 | 59215 | 26503 | 11060 | 8924 | 6280 | 699 | 692 | 23 | 24 |
|  | 19 | 60865 | 27620 | 7984 | 6513 | 4126 | 486 | 494 | 27 | 27 |
