# Supplementary material for: Next-Generation Sequencing of Aquatic Oligochaetes: Comparison of Experimental Communities
Source: PLoS One. 2016 Feb 11;11(2):e0148644. doi: 10.1371/journal.pone.0148644 (PMC4750909; doi:10.1371/journal.pone.0148644)
Supplement: S4 Table — (DOC) [file pone.0148644.s006.doc]

| OTU | S1 | S2 | S3 | S4 | S5 | S6 |
| --- | --- | --- | --- | --- | --- | --- |
| *Bothrioneurum vejdovskyanum* R1 | NA | 122 | 123.44 | 66.63 | NA | NA |
| Enchytraeidae sp. (1) | 4.31 | NA | NA | NA | NA | NA |
| Enchytraeidae sp. (2) | NA | NA | NA | NA | NA | 7 |
| Indet (1) | NA | NA | NA | NA | NA | NA |
| Indet (2) | NA | NA | NA | NA | NA | NA |
| *Limnodrilus claparedeanus* T22 | 1.17 | 0.45 | 0.41 | 4.85 | NA | 1.17 |
| *Limnodrilus hoffmeisteri* T17 | 1.11 | 0.75 | 0.56 | 0.8 | 1.89 | 0.91 |
| *Limnodrilus hoffmeisteri* T18 | 3.68 | NA | NA | 1.17 | NA | 0.85 |
| *Limnodrilus hoffmeisteri* T19 | 1.82 | 1.07 | NA | NA | NA | NA |
| *Limnodrilus hoffmeisteri* T20 | 2.17 | NA | 0.71 | NA | NA | 4.9 |
| *Limnodrilus hoffmeisteri* T21 | 0.3 | 0.41 | 0.26 | NA | NA | 0.31 |
| *Limnodrilus udekemianus* T23 | NA | NA | NA | 26.6 | NA | NA |
| *Lumbricillus rivalis* E3 | NA | NA | NA | 25 | 51.31 | NA |
| *Marionina* sp. (1) | NA | NA | 185 | NA | NA | NA |
| *Marionina* sp. (2) | 1.55 | 4 | 1.11 | NA | NA | NA |
| *Nais elinguis* N4 | NA | NA | NA | NA | NA | 5.46 |
| *Psammoryctides barbatus* T8 | 2.19 | 0.76 | NA | NA | NA | 0.9 |
| *Potamothrix bavaricus* T7 | 1.08 | 1.54 | 0.74 | NA | NA | 1.5 |
| Tub. with hair setae (1) | 0.86 | NA | NA | NA | NA | NA |
| Tub. with hair setae (2) | NA | NA | NA | 1.27 | NA | NA |
| Tub. with hair setae T2 | NA | NA | NA | NA | NA | 2.06 |
| Tub. with hair setae T3 | NA | NA | NA | NA | NA | 7.26 |
| Tub. without hair setae (1) | 6.1 | NA | NA | NA | NA | NA |
| Tub. without hair setae (2) | NA | NA | NA | NA | NA | NA |
| Tub. without hair setae (3) | NA | NA | NA | NA | NA | NA |
| Tub. without hair setae (4) | NA | NA | NA | NA | NA | NA |
| Tub. without hair setae T14 | NA | 0.6 | NA | NA | NA | NA |
| Tub. without hair setae T15 | 214.29 | 104.54 | 137.19 | 88.89 | 83.25 | 122.5 |
| Tub. without hair setae T16 | NA | NA | NA | 1.35 | 19.59 | NA |
| Tub. without hair setae (5) | NA | NA | NA | 3.31 | 2.78 | NA |
| Tub. without hair setae (6) | NA | NA | NA | NA | NA | NA |
| *Lophochaeta ignota* T6 | 0.46 | 0.64 | NA | NA | NA | 0.75 |
| *Tubifex tubifex* T10 | NA | NA | NA | NA | 0.41 | NA |
| *Tubifex tubifex* T11 | NA | NA | NA | 2.8 | 1.96 | 7.54 |
| *Tubifex tubifex* T9 | NA | NA | 0.59 | 0.46 | 0.45 | NA |

NA : comparison of proportions impossible due to the absence of the simultaneous presence of specimen and sequence ; Indet = unidentified
